# Inhibitory Effect of *Trichoderma longibrachiatum* on Growth of *Fusarium* Species and Accumulation of Fumonisins

**DOI:** 10.3390/jof12010049

**Published:** 2026-01-10

**Authors:** Ruiqing Zhu, Ying Li, María Viñas, Qing Kong, Manlin Xu, Xia Zhang, Xinying Song, Kang He, Zhiqing Guo

**Affiliations:** 1 Shandong Peanut Research Institute, Qingdao 266100, China; zhuruiqing0311@foxmail.com (R.Z.); li1989ying0921@163.com (Y.L.); xumanlin@126.com (M.X.);; 2 National Engineering Research Center for Peanut, Qingdao 266100, China; 3 School of Food Science and Engineering, Ocean University of China, Qingdao 266404, China; kongqing@ouc.edu.cn; 4 Center for Research in Grains and Seeds (CIGRAS), University of Costa Rica, San Pedro, San José 11501, Costa Rica; maria.vinasmeneses@ucr.ac.cr

**Keywords:** *Trichoderma longibrachiatum*, *Fusarium* spp., fumonisins, biological control

## Abstract

*Fusarium* spp. cause devastating crop diseases and produce carcinogenic mycotoxins such as fumonisins, threatening global food safety and human health. In this study, *Trichoderma longibrachiatum* A25011, isolated from apples in Aksu, Xinjiang, exhibited significant antagonistic activity with mycelial growth inhibition rates of 54.52% against *F. verticillioides* 48.62% against *F. proliferatum*, and 58.22% against *F. oxysporum* in confrontation assays. Enzyme activity detection revealed high chitinase (583.21 U/mg protein) and moderate cellulase (43.92 U/mg protein) production, which may have the capacity to degrade fungal cell walls. High-Performance Liquid Chromatography–Mass Spectrometry (HPLC-MS/MS) analyses enabled the quantification of fungal hormones including gibberellin A3 (GA3, 2.44 mg/L), cytokinins (cis-zeatin riboside (CZR): 0.69 mg/L; trans-zeatin riboside (TZR): 0.004 mg/L; kinetin: 0.006 mg/L), and auxins (indole-3-acetic acid (IAA): 0.35 mg/L; abscisic acid: 0.06 mg/L). Application of a *T. longibrachiatum* A25011 spore suspension around the roots of peanut plants enhanced growth by 13.20% (height), 5.65% (stem and leaf biomass), and 39.13% (root biomass). Notably, A25011 reduced *F. proliferatum*-derived fumonisin accumulation in rice-based cultures by 93.58% (6 d) and 99.35% (10 d), suggesting biosynthetic suppression. The results demonstrated that *T. longibrachiatum* strain A25011 exhibited excellent biocontrol capability against *Fusarium* spp., proving its dual role in simultaneously suppressing fungal growth and fumonisin accumulation while promoting plant growth. *T. longibrachiatum* A25011 could be applied as a multifunctional biocontrol agent in sustainable agriculture in the future.

## 1. Introduction

*Fusarium* species are important phytopathogenic fungi that infect a wide range of agricultural crops, causing diseases including root rot, stem rot, and fruit rot. Root rot is mainly characterized by a “rat-tail”-like rot of the root system. In severe cases, it can cause seedling death at maturity [1]. Stem rot is caused by the combined infection of *Fusarium* spp. and *Pythium* spp. on the vascular bundles at the base of plant stems. The infection blocks water transport, producing dark brown lesions and ultimately leading to the entire plant wilting [2,3]. ‌Fruit rot is caused by *Fusarium* spp. infecting fruits or seeds and disrupting seed development or causing seed rot [4,5]. *Fusarium* spp. infection not only diminishes crop yield but also reduces the quality of crop fruits or seeds (e.g., appearance and nutritional value).

‌Furthermore, grains can be contaminated by *Fusarium* spp., which produce mycotoxins that pose risks to human and animal health when ingested [6,7]. The principal mycotoxins produced by *Fusarium* spp. include fumonisins (FB), vomitoxin (deoxynivalenol, DON), and zearalenone (ZEN) [8,9]. Fumonisins are mainly produced by *F*. *verticillioides* and *F*. *proliferatum*. The chemical structures of many different fumonisin groups (FA1, FA2, FA3, FB1, FB2, FB3, FB4, FP1, FP2, and FP3) have been identified [10]. The B group exhibits strong toxicity, among which fumonisin B1 (FB1) is the most abundant and toxic. Its hepatotoxicity and carcinogenicity have been verified in rat experiments [8,11,12]. It was simultaneously associated with ‌Equine Leukoencephalomalacia (ELEM)‌ and induced an imbalance of neurotransmitters in the human body by interfering with sphingolipid metabolism, exhibiting strong neurotoxicity [13,14]. Fumonisin B1 has been classified as a Group 2B carcinogen (possibly carcinogenic to humans) by the International Agency for Research on Cancer [15].

‌Traditional prevention and control of phytopathogenic fungi primarily rely on resistant cultivar breeding, integrated environmental control, physical processing, and chemical detoxification.‌ However, these approaches present significant limitations. Resistance breeding involves substantial technical challenges and prohibitive developmental costs. Environmental control exhibits stochastic variability and poor operational predictability. Physical processing causes nutrient loss in crops, and chemical detoxification poses risks of secondary toxicity to human beings [16,17]. In contrast, ‌biological control demonstrates environmental compatibility, favorable safety profiles, and enhanced efficacy‌. This approach plays a crucial role in mitigating plant diseases while preserving natural ecosystem equilibrium, positioning it as one of the most promising sustainable management strategies [16]. Among microbial biological control agents (BCAs), *Trichoderma* species represent one of the most extensively commercialized and agronomically significant for biocontrol applications [18,19].

The fungi *Trichoderma* spp. (*Ascomycota*: *Hypocreales*) ubiquitously colonize soil and rhizosphere ecosystems [20]. Representative species, such as *T. harzianum*, *T. longibrachiatum*, *T. atroviride*, *T. viride*, and *T. asperellum*, are commonly found in nature. They can rapidly establish soil colonization and enhance crop performance through dual mechanisms of growth promotion and phytopathogen suppression, demonstrating a symbiotic relationship that is beneficial to plants [21].

‌Recently, many researchers have elucidated that most *Trichoderma* species exhibited antagonistic effects against peanut (*Arachis hypogaea*) pathogens. Their antagonistic ability was a combination of multiple mechanisms, including nutrient competition, direct parasitism of pathogenic fungi [20,22,23], release of elicitors to induce disease resistance in crops, and production of secondary metabolites to inhibit the growth of pathogens [24,25,26,27]. Therefore, *Trichoderma* species are globally deployed as biological control agents and soil fertilizer enhancers worldwide [28]. Furthermore, studies have demonstrated their capacities to reduce mycotoxin contamination through various mechanisms in agricultural systems, thereby mitigating associated human health risks [29]. Among diverse *Trichoderma* species, *T*. *longibrachiatum* exhibits significant biocontrol research potential characterized by accelerated mycelial proliferation, robust sporulation capacity, and broad-spectrum antagonistic activity against plant pathogens. Li et al. reported that fermented filtrates of *T. longibrachiatum* strain SC5 effectively suppressed *Sclerotinia sclerotiorum* mycelial growth and germination [30]. Wang identified effector protein TLCpe1 from strain SMF2 that enhanced host disease resistance [31].

‌In this study, a *Trichoderma* strain exhibiting rapid radial expansion and antagonistic halos against neighboring microorganisms was isolated from the surface of Xinjiang Aksu apple fruits. It was identified as *T. longibrachiatum* by morphological characteristics and molecular methods. Apple as a host for *T. longibrachiatum* was rarely reported. The strain, named A25011 by our team, showed significant inhibition of *F. proliferatum* and *F. verticillioides* mycelial growth and reduced the accumulation of fumonisins by *F. proliferatum*. Here, we reported that *T. longibrachiatum* inhibited fumonisin accumulation. Bioactive metabolites, including hormones and enzymes underlying plant growth promotion in peanut (*Arachis hypogaea*) activities, were characterized. This investigation demonstrated the dual efficacy of *T. longibrachiatum* A25011 in enhancing plant growth and reducing mycotoxin accumulation.

## 2. Materials and Methods

### 2.1. Isolation and Purification of Fungal Antagonist from Apples

The collection of surface microorganisms from Xinjiang Aksu apples was conducted using a sterile brush pre-dipped in autoclaved distilled water. The brush was gently swabbed across the stem and epicarp to collect the microorganisms. The suspension (50 μL) was evenly spread onto a Potato Dextrose Agar (PDA) plate and incubated at 28 °C under dark condition for 48 h to allow fungal growth. Plates were examined for characteristic Trichoderma-like colonies exhibiting rapid radial growth and inhibitory halos against neighboring microbiota. Morphologically distinct presumptive *Trichoderma* strains were selected and subjected to single-spore purification to obtain axenic cultures.

### 2.2. Molecular Identification of Fungal Antagonist

**Figure 1:**
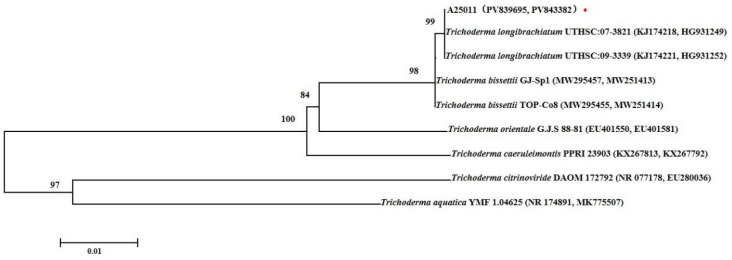
The phylogenetic tree of the strain A25011 according to the sequence analysis of ITS/TEF with the neighbor-joining approach. The strain marked with a red dot is A25011. Percentages refer to the bootstrap values using 1000 repetitions.

The purified fungal colonies exhibiting morphological characteristics [32] consistent with *Trichoderma* spp. were subjected to genomic deoxyribonucleic acid (DNA) extraction with the cetyl trimethyl ammonium bromide (CTAB) method. The internal transcribed spacer (ITS) region was amplified using the primers ITS1 (5′-TCCGTAGGTGAACCTGCGG-3′) and ITS4 (5′-TCCTCCGCTTATTGATATGC-3′) [33], with the polymerase chain reaction (PCR) fragments sequenced and subjected to Basic Local Alignment Search Tool (BLAST, 2.17.0) analysis against the NCBI database for preliminary species identification. For phylogenetic resolution improvement, the translation elongation factor 1-alpha (TEF1) gene was amplified with species-specific primers EF1-728 (5′-CATCGAGAAGTTCGAGAAGG-3′) and TEF1R (5′-GCCATCCTTGGGAGATACCAGC-3′) [34], with the PCR products subsequently sequenced and analyzed through NCBI sequence comparisons. The phylogenetic tree combining ITS and TEF sequences is shown in Figure 1. The phylogenetic tree was constructed using neighbor-joining analysis (the maximum composite likelihood model in MEGA 7.0) generated from ITS and TEF sequences of strain A25011 from this study and other *Trichoderma* spp. from GenBank. The numbers on the branch points represent bootstrap values of the tree (1000 replicates). The scale bar represents a genetic distance of 0.01 horizontally.

### 2.3. Confrontation Assays Between Fungal Antagonist and Fusarium spp.

**Figure 2:**
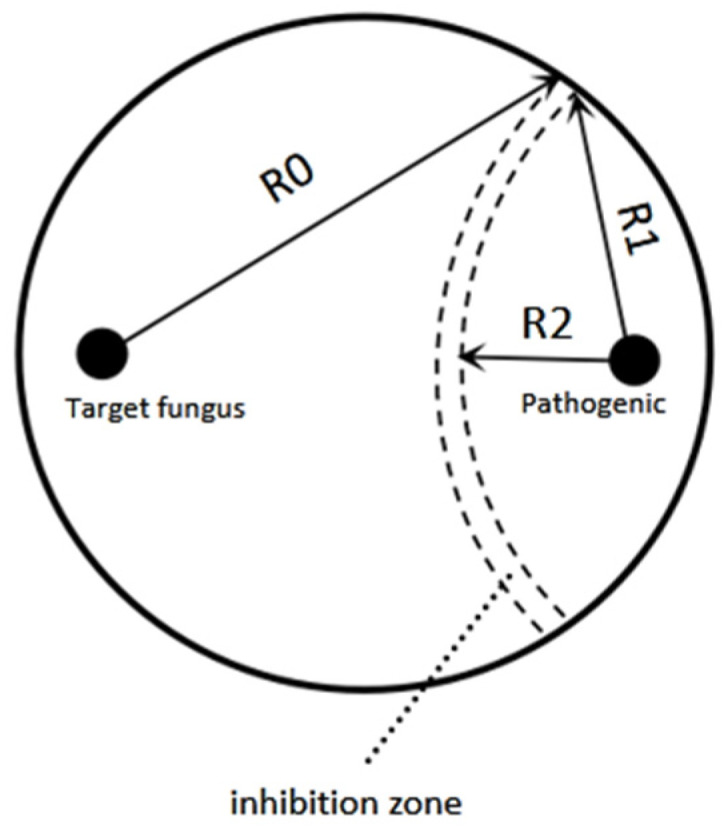
The figure was drawn according to Ren [35]. R0 is the growth radius of the antagonist, R1 is the growth radius of the pathogen that is not inhibited, and R2 is the growth radius of the pathogen that is inhibited by the antagonist. The dashed area in the figure represents the potential inhibition zone in the confrontation plate, where neither of the two microorganisms grows.

The antagonistic activity of *T. longibrachiatum* A25011 was evaluated by confrontation assays. PDA plates were inoculated with 5 mm mycelial disks from actively growing cultures of *F. verticillioides* (isolated from infected maize kernels and kindly provided by associate professor Guangfei Tang from Institute of Plant Protection, Chinese Academy of Agricultural Sciences), *F. proliferatum* (isolated from infected maize kernels and identified by our lab), or *F. oxysporum* (isolated from infected peanut root and identified by our lab) and the target fungal antagonist. In the test plates, paired inoculations were performed: *F. verticillioides, F. proliferatum,* or *F. oxysporum* disks were positioned at one edge of the plate, while antagonist disks were placed symmetrically at the opposite edge. Group A contained only the *Fusarium* spp. pathogen, whereas the control contained only the antagonist. Three independent replicates were performed, and three Petri dishes were performed within each replicate. Test and control plates were incubated at 28 °C for 96 h in darkness. Mycelial growth was measured, and the inhibition rate of the pathogenic fungi was calculated [35] as shown in Figure 2.

$$\mathrm{Radial}\;\mathrm{growth}\;\mathrm{inhibition}\;\mathrm{rate}’\;\mu =\frac{\left(R1-R2\right)}{R1}\times 100\%$$

### 2.4. Pot Experiments on the Effects of F. oxysporum and Fungal Antagonists on Peanut Growth 

Pot experiments were conducted to evaluate the biocontrol effects of strain *T. longibrachiatum* A25011 on peanut (*Arachis hypogaea* cv. Zhonghua 12) plant growth. Seeds were divided into 4 groups: untreated control (CK), *F. oxysporum*-inoculated (Fo), *T. longibrachiatum* A25011-inoculated (A), and *T. longibrachiatum* A25011 + *F. oxysporum* co-inoculation (A + Fo). Seeds of all groups were sown in autoclaved soil and maintained at 24 °C, under constant humidity and continuous illumination.

Peanut seedlings (7 days old) of groups A and A + Fo were inoculated at the base with 5 mL/plant of *T. longibrachiatum* A25011 spores (10^6^ spores/mL). We prepared the spore suspension in advance and calculated the spore concentration using a hemocytometer. Peanut seedlings (14 days old) of groups Fo and A + Fo were inoculated with 5 mL/plant *F. oxysporum* (isolated from the peanut rot root in 2017 in China) with a spore suspension of 10^6^ spores/mL at the base of the plant. Phenotypic assessments on the 28th day were recorded, including (I) stem and leaf fresh weight, (II) root fresh weight, and (III) plant height across all treatment groups according to Li et al. [30]. Three independent replicates were performed, each with 24 plants divided into 4 groups. The experimental unit was one plant per pot.

### 2.5. Determination of Fungal Hormones

#### 2.5.1. Sample Processing for Hormone Assay

The mycelium (7 days old) of *T. longibrachiatum* A25011on PDA plates was collected, snap-frozen in liquid nitrogen, and immediately ground into powder. Three independent replicates were performed. Precise aliquots (0.2 g) of powder were measured using an electronic balance (ML-204, Toledo, OH, USA) and placed into sterile glass test tubes. For hormone extraction, 7 mL of the extraction solution of isopropanol–water–hydrochloric acid (2:1:0.002 *v*/*v*) and 8 µL (1 µg/mL) of the internal standard solution of each target hormone were sequentially added to the glass test tube, and the test tube was vibrated for 30 min at 4 °C. A total of 8 mL of Dichloromethane was added, and the test tube was shaken for an additional 30 min at 4 °C. After shaking, the mixture was centrifuged in a high-speed centrifuge (TGL-20bR, Shanghai, China) at 13,000 rpm for 5 min at 4 °C. The lower organic phase was collected, dried using a nitrogen evaporator (DC150-2, Hangzhou, China), and then redissolved in 400 μL of methanol (containing 0.1% formic acid) under dark conditions. The solution was centrifuged at 13,000 rpm for 10 min at 4 °C, and the supernatant was collected and filtered through 0.22 µm membranes prior to HPLC-MS/MS analysis.

#### 2.5.2. HPLC-MS/MS Analyses of Fungal Hormones

Chromatographic separation was performed using a Vanquish HPLC system (Waltham, MA, USA) equipped with a Syncronis aQ column (100 × 2.1 mm, 1.7 µm particle size) maintained at 40 °C. Mobile phase A included 0.1% formic acid in water, while mobile phase B included methanol (containing 0.1% formic acid). The time gradient was 0, 5, 10, 10.6, 15, 15.01, and 18 min (A%: 98, 98, 50, 100, 98, and 98; B%: 2, 2, 50, 0, 2, and 2). Separation parameters included a 5 µL injection volume and 0.3 mL/min flow rate. After separation, analytes were introduced into a TSQ Altis Plus mass spectrometer (Waltham, MA, USA) operating in positive/negative switching modes with electrospray ionization (ESI). MS detection employed multiple reaction monitoring (MRM) with the following parameters: spray voltages of 3500 V (positive ion mode) and 2500 V (negative ion mode), a sheath gas flow rate of 60 Arb, an auxiliary gas flow rate of 15 Arb, an ion transfer tube temperature of 380 °C, and a vaporizer temperature of 350 °C. Calibration curves were established for all target hormones (Table 1).

### 2.6. Enzyme Activity Determination for T. longibrachiatum A25011

#### 2.6.1. Sample Processing for Enzyme Activity Assay

Fungal biomass (including mycelium and spores) was gently scraped from PDA plates (7 days old) and suspended in 0.01 M phosphate-buffered saline (pH = 7.4). Three independent replicates were performed. Cellular extraction was performed using a tissue homogenizer with an ice bath, followed by centrifugation at 8500 rpm for 10 min at 4 °C (TGL-20bR high-speed centrifuge, Shanghai, China). The supernatant was collected for fungal enzyme activity determination.

#### 2.6.2. Determination of Chitinase

The assay principle is that chitinase hydrolyzes the chitin substrate to generate N-acetylglucosamine. The intermediate compound produced by heating N-acetylglucosamine with alkali can subsequently react with p-dimethylaminobenzaldehyde to form a chromogenic substance exhibiting a characteristic absorption maximum at 585 nm. The chitinase activity is quantified by measuring absorbance changes at this wavelength using a commercial chitinase assay kit (Nanjing, China).

For the different experiment treatments, 100 μL of the sample extract, 300 μL of reagent 1, and 600 μL of reagent 2 were sequentially added to the tubes respectively. For the control, 100 μL of the sample extract, 300 μL of reagent 1, and 600 μL of distilled water were sequentially injected into the tubes. After thoroughly mixing, tubes were incubated at 37 °C for 30 min in a digital constant-temperature water bath (HH-6, Changzhou, China). Centrifugation was then performed at 10,000 rpm for 10 min at room temperature (TGL-20bR centrifuge, Shanghai, China). Subsequently, 250 μL of the supernatant from each tube was transferred to new tubes. In each tube, 250 μL of distilled water and 250 μL of 50 μg/mL standard solution were added. In all tubes, 50 μL of reagent 3 was added, and the solution was mixed and heated in a water bath for 5 min, followed by cooling to room temperature under running water. In the tubes, 750 μL of reagent 4 was added and mixed, and the tubes were incubated at 37 °C for 20 min. Absorbance was measured at 585 nm in 1 mL glass cuvettes using a spectrophotometer (UV5800H, Shanghai, China) zeroed with distilled water.

Here, 1 unit of chitinase activity was defined as the amount of enzyme required to liberate 1 μg of N-acetylglucosamine from chitin per mg of protein per minute under specified assay conditions. The enzyme activity was calculated according to the formula:

U (mg protein) = [(ΔA1 ÷ ΔA2 × C1) × V1] ÷ (V2 × C2) ÷ T

ΔA1 denotes the absorbance difference between test and control samples, ΔA2 represents the absorbance difference between standard and blank samples, C1 indicates the standard concentration (50 μg/mL), V1 is the total reaction volume (1 mL), T signifies the reaction time (30 min), C2 refers to the protein concentration (mg/mL), and V2 signifies the sample volume (0.1 mL). Specific enzyme activity values are quoted as units/mg protein, which indicates how many units of enzyme activity are contained per milligram of total protein.

#### 2.6.3. Cellulase Activity Determination

Reducing sugar content derived from cellulose degradation by cellulase was quantified using the 3,5-dinitrosalicylic acid (DNS) method with a Cellulase Assay Kit (Nanjing, China). Briefly, 50 μL of the sample solution and 150 μL of reagent 1 were added to the tube, while 50 μL of the sample solution and 150 μL of distilled water were added to the tube for the control. Furthermore, 50 μL of the standard solution (graded concentrations) and 150 μL of distilled water were added to the tube as standards, and 150 μL of distilled water was added to the tube as a blank control. Different samples were thoroughly mixed and incubated at 50 °C in a water bath (HH-6, Changzhou, China) for 30 min. Subsequently, 150 μL of reagent 2 was added, thoroughly mixed, and boiled for 5 min. After samples of different treatments were cooled down to room temperature, 1000 μL of distilled water was added and evenly mixed. Absorbance was measured using a spectrophotometer at 540 nm (UV5800H, Shanghai, China) for standard (A1), test (A3), control (A3), and blank (A0) treatments. The absorbance for standards was calculated as ΔA1 = A1 − A0, while the test sample absorbance was ΔA2 = A2 − A3. A standard curve was established by plotting standard concentrations (y, mg/mL) against ΔA_1_ values (x). Sample concentration C (y, mg/mL) was determined by substituting ΔA2 (x) into the standard curve equation. One unit of cellulase activity (U/mg protein) was defined as the amount of enzyme required to catalyze the release of 1 μg of glucose per mg of protein per minute. Enzyme activity was calculated as follows:

U (mg protein) = 1000 × C × V ÷ (V × Cpr) ÷ T

where V denotes sample volume (0.05 mL), T represents reaction time (30 min), and Cpr indicates protein concentration (mg/mL).

### 2.7. Inhibition of F. proliferatum Mycotoxin Accumulation by T. longibrachiatum A25011

Rice kernels (cultivar Daohuaxiang, Wuchang, China) were immersed in sterile water overnight, and 20 g of kernels was placed into 100 mL Erlenmeyer flasks containing 5 mL of sterile water before autoclaving to prepare rice-based media. Four treatments were conducted: A (only the antagonist), A + Fp (the antagonist and *F. proliferatum*), Fp (only *F. proliferatum*), and CK (the negative control). Spore suspensions (10^6^ spores/mL) of *F. proliferatum* (Fp) and biocontrol fungus *T. longibrachiatum* A25011 were prepared. Autoclaved rice kernels were inoculated with 1 mL of *T. longibrachiatum* A25011 (A) or 1 mL of *F. proliferatum* conidia suspension. Test autoclaved rice kernels were inoculated with 1 mL of a *T. longibrachiatum* A25011 and *F. proliferatum* conidia suspension (A + Fp). Negative control (CK) rice was handled in the same way without any conidia inoculation [36]. All rice samples were incubated at 28 °C for 10 d. Three replicates per treatment were performed. After 10 d, the rice cultures were collected and stored in −20 °C for fumonisin quantification.

Rice samples of different groups prepared above were freeze-dried and ground into a powder, and fumonisins were analyzed with an enzyme-linked immunosorbent assay (ELISA) kit (Beijing, China) according to the manufacturer’s instructions. Extracts of all groups were centrifuged (4000 rpm, 10 min), and supernatants were collected: the Fp group supernatant was diluted 100-fold, the A + Fp group supernatant was diluted 10-fold, and A and CK group supernatants were without dilution. After a second dilution (0.5 mL supernatant + 4.5 mL deionized water), 50 μL aliquots were analyzed in microplate wells assigned to standards and samples (duplicated), with positions recorded. Standards/samples (50 μL/well), fumonisin–enzyme conjugates (50 μL/well), and fumonisin antibodies (50 μL/well) were added sequentially, mixed gently, sealed, and incubated (25 °C, dark, 15 min). After decanting, wells were washed (250 μL/well wash buffer, 4×), blotted dry, and incubated with substrate (100 μL/well, 25 °C dark, 5 min); stop solution (50 μL/well) was then added and mixed, and the intensity of the resulting yellow color was measured using a 96-well iMark™ Microplate Absorbance Reader (BIO-RAD, Hercules, CA, USA) at a 450 nm filter with a differential filter of 630 nm. The OD values of the five fumonisin standard concentrations were constructed as a dose–response curve, and concentration in the unknowns was measured by interpolation from this standard curve. The results were calculated using Ridawin.NET provided by Huaan Magnech Bio-Tech, CN. And the correlation coefficient (R^2^) of the calibration curve was between 0.990 and 1.000 [37]. The limits of quantification for fumonisins ranged between 5 μg/kg and 250 μg/kg. The fumonisin inhibition rate (*μ*) was calculated as follows:

$$\mu =(1-\frac{D1-D0}{D2-D0})\times 100\%$$

where D1 = A + Fp, D2 = Fp, and D0 = CK (untreated control).

### 2.8. Statistical Analysis

Statistical analyses were conducted with Statistical Product and Service Solutions (SPSS) 23.0. Means and standard deviations (S.D.s) are given in the bar graphs. Confrontation assays, fresh weight of stems, leaves, and roots, and plant height of peanut seedling data were analyzed by an ANOVA, and Duncan’s multiple range test was used to calculate the significant differences between each group. The experiments used a completely randomized design (CRD). The replicate number and the concentration of spores per replicate were specified in the description of the experiments in the Materials and Methods Section.

## 3. Results

### 3.1. Isolation and Identification of T. longibrachiatum A25011

Sixty morphologically different, rapidly growing fungal colonies were independently isolated from apple fruits and purified within 48 h on PDA cultures. Among these sixty strains, six were identified as *Trichoderma* species. The fungus with the fastest growth rate and the ability to inhibit surrounding microorganisms was marked as A25011, which was selected for morphological characterization, molecular identification, phylogenetic analysis, and further experiments to determine its biocontrol properties. On PDA, colonies initially appeared white, dense, and circular; on day 2, they developed an abundantly long, white, and fluffy aerial mycelium; on day 3, the mycelium rapidly expanded to cover 9 cm plates with green conidia production in central regions forming distinct concentric rings; and by day 5, the entire colony turned green. For molecular identification, ITS sequence analysis (GenBank accession No. PV839695) resulted in a 100% match with *T. longibrachiatum* T-KN3 (GenBank accession No. LT707585) by BLAST analysis in the NCBI nucleotide database. The TEF sequences (GenBank accession No. PV843382) showed 100% identity with *T. longibrachiatum* SzMC (EU401624). The phylogenetic analysis based on ITS and TEF sequences was constructed using the neighbor-joining algorithm of MEGA7.0 (Figure 2). Based on the above data, the strain A25011 was identified as *T. longibrachiatum*.

### 3.2. Confrontation Assay Between Fungal Antagonist and Fusarium spp.

**Figure 3:**
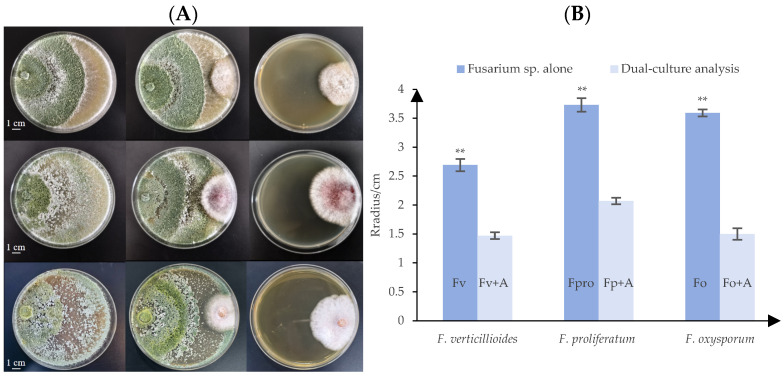
Confrontation assays. (**A**) Experimental confrontation among *F. verticillioides* (**top**), *F. proliferatum* (**middle**), and *F. oxysporum* (**bottom**) and *T. longibrachiatum* A25011 on agar plates: from left to right are *Trichoderma* sp. alone, dual culture, and *Fusarium* sp. alone. (**B**) Comparison of radii between experimental and control groups of two types of *Fusarium* species. Data were analyzed by an ANOVA (** *p* < 0.01). Means and standard deviations (S.D.s) are given in the bar graphs (n = 108). Measurements after 96 h.

The confrontation assay quantified the inhibitory effects of A25011 on mycelia growth across *Fusarium* species, confirming antagonistic activity against *F. verticillioides*, *F. proliferatum*, and *F. oxysporum* (Figure 3A). Inhibition zones were virtually undetectable between *T. longibrachiatum* and the test pathogens, with distal mycelia of *T. longibrachiatum* exhibiting aggressive orientation toward *Fusarium* colonies indicative of mycoparasitism. After 96 h, mycelial radial growth was significantly reduced when *F. verticillioides*, *F. proliferatum*, or *F. oxysporum* were confronted against *T. longibrachiatum* strain A25011 (Figure 3B, *p* < 0.01), yielding growth inhibition rates of 54.52% against *F. verticillioides*, 48.62% against *F. proliferatum*, and 58.22% against *F. oxysporum*.

### 3.3. Effects of Fungal Antagonist on Peanut Growth

**Figure 4:**
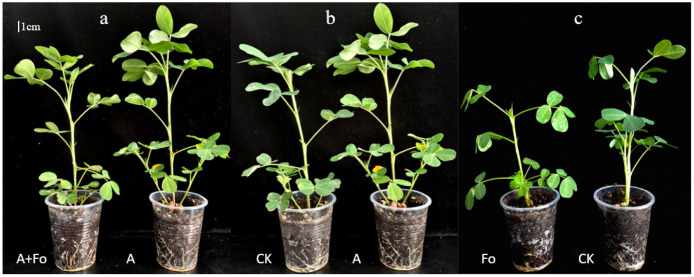
Comparison of peanut plant growth between Group A + Fo and Group A (**a**), Group CK and Group A (**b**), and Group Fo and Group CK (**c**); 28 days post-inoculation with the antagonist *T. longibrachiatum* A25011.

**Figure 5:**
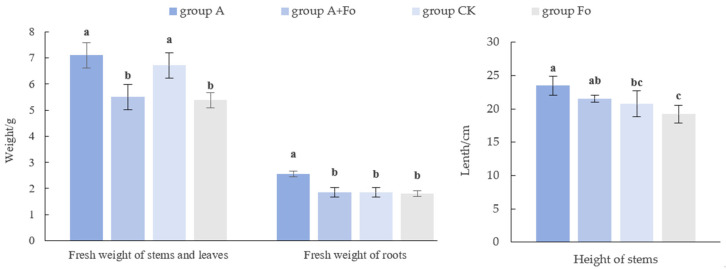
Fresh weight of stems, leaves, and roots and plant height of peanut seedlings 28 d post-inoculation with antagonist *T. longibrachiatum* A25011 (Group A), the antagonist and pathogen *F. oxysporum* (Group A + Fo), the untreated control (Group CK), and only the pathogen (Group Fo). Letters a, b, and c mean significant differences among each other (*p* < 0.05), while ab means a significant difference with c and bc means a significant difference with a. Data were analyzed by an ANOVA. Means and standard deviations (S.D.s) are given in the bar graphs (n = 72).

Compared to the control, *T. longibrachiatum* A25011 exhibited significant growth promotion in peanut plants after 28 d post-inoculation (Figure 4 and Figure 5). For instance, the peanut plant height and shoot and root fresh weight of the plants inoculated only with *T. longibrachiatum* A25011 (Group A) were 13.20%, 5.65%, and 39.13% higher than in the control, respectively (Group CK). These results confirmed the multifaceted growth-promoting effects of *T. longibrachiatum* A250111. In contrast, *F. oxysporum*-inoculated (Fo plants) showed marked growth suppression with 23.18% shoot fresh weight reduction and concurrent decrease in other parameters when compared to Group CK. All parameters of peanut plants inoculated with *T. longibrachiatum* A25011 (Group A) were higher than those of Group Fo and showed a significant difference (*p* < 0.05). Interestingly, co-inoculated plants (the A + Fo group) exhibited growth (weight) parameters restored to CK-equivalent levels, demonstrating *T. longibrachiatum*’s antagonistic capacity against *F. oxysporum* infection.

### 3.4. Determination of Hormones and Enzyme Activity Assays of the Antagonist T. longibrachiatum A25011

Hormone quantification in *T. longibrachiatum* strain A25011 (Table 2) was performed using HPLC-MS, revealing GA3 as the most abundant endogenous growth regulator at 2.44 mg/L, a concentration within the physiologically active range that promotes seed germination. Both CZR and TZR were detected, and TZR had a lower concentration (0.004 mg/L) compared to CZR (0.69 mg/L). IAA, the principal auxin, was quantified at 0.35 mg/L, and the concentration of ABA was at a low level (0.06 mg/L). KT was detected at minimal levels, suggesting only synergistic roles in this strain.

Enzyme activity (Table 3) assays for chitinase and cellulase revealed that *T. longibrachiatum* A25011 exhibited high chitinase activity (583.21 mgprot) and moderate cellulase activity (43.92 mgprot). Given that fungal cell walls are mainly composed of chitin and cellulose, these findings demonstrated that *T. longibrachiatum* A25011 could parasitize and inhibit *Fusarium* growth primarily through enzyme degradation of its cell-wall components.

### 3.5. Inhibition of Fumonisin Production by T. longibrachiatum Strain A25011

**Figure 6:**
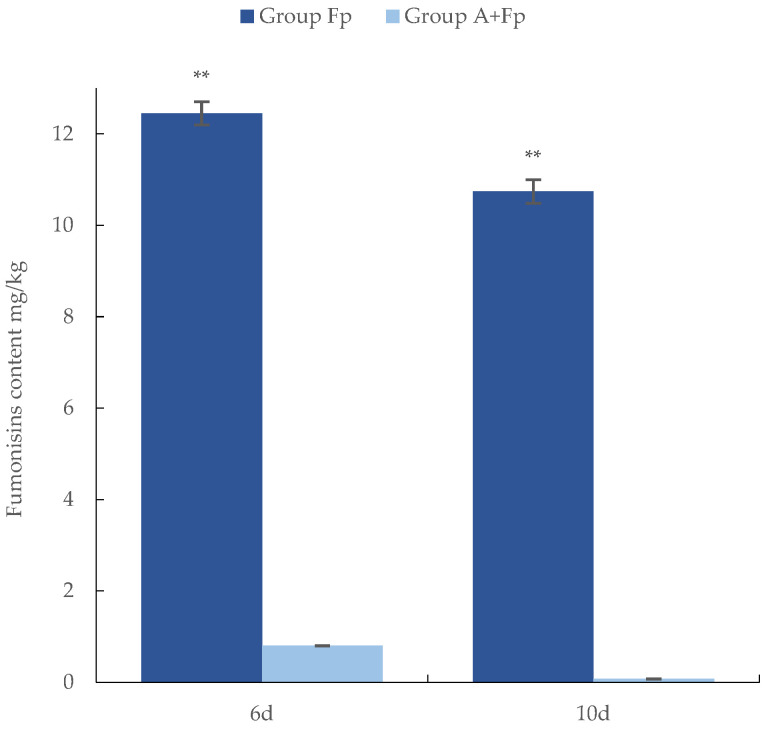
Concentration of fumonisins in rice kernels inoculated with only *F. proliferatum* (group Fp) or with *F. proliferatum* and the antagonist *T. longibrachiatum* A25011 (Group A + Fp). Samples were collected on days 6 and 10. Data were analyzed by an ANOVA (** *p* < 0.01). Means and standard deviations (S.D.s) are given in the bar graphs (n = 48).

Fumonisin production by *F. proliferatum* was significantly reduced after 6 and 10 days of co-cultivation with the antagonist *T. longibrachiatum* strain A25011 on rice cultures (Figure 6). As expected, *F. proliferatum* untreated groups (A and CK) showed undetectable levels (0 mg/kg) on both days. When the pathogen was cultivated alone (group Fp), high concentrations of the toxin were produced after 6 and 10 days, with 12.45 mg/kg and 10.74 mg/kg of fumonisins, respectively. However, when it was cultivated with the antagonist (Group A + Fp), the concentration of fumonisins was significantly lower (0.80 mg/kg and 0.07 mg/kg at corresponding timepoints). The calculated inhibition rates (η) of *T. longibrachiatum* A25011 reached 93.58% (η6d) and 99.35% (η10d), confirming its potent suppression of *F. proliferatum* toxigenesis. Notably, the 11-fold decrease in fumonisin levels between days 6 and 10 in Group A + Fp significantly exceeded the minor reduction observed in group Fp (1.16-fold).

## 4. Discussion

Our study investigated the biocontrol mechanisms of the *T. longibrachiatum* A25011 strain against *Fusarium* spp., with particular emphasis on its dual functions in promoting plant growth and inhibiting fungal growth and mycotoxin production. *F. verticillioides* and *F. proliferatum* were both isolated from infected maize kernels, and the capacities of fumonisin production were confirmed. In plate confrontation assays, *T. longibrachiatum* A25011 exhibited significant inhibition of *F. verticillioides*, *F. proliferatum* and *F. oxysporum* mycelial growth and displayed aggressive parasitic behavior towards the pathogen. *F. oxysporum* was originally isolated from the rot root of peanut in Shandong Province and rarely produced fumonisins. We used it for the root infection experiments. Previous research has demonstrated that *Trichoderma* spp. have the capacity to produce secondary metabolites and cell-wall-degrading enzymes to inhibit pathogens [38,39].

Enzyme activity assays revealed that the inhibition of the pathogen growth caused by *T. longibrachiatum* A25011 could potentially be due to enzyme degradation of fungal cell walls, with notably high chitinase and cellulase activities targeting the chitin and cellulose structures of *Fusarium* mycelia, thereby disrupting their cellular structure. Previous research has demonstrated a positive correlation between chitinase activity and antagonistic effects [40]. Additionally, most *Trichoderma* strains have strong decomposition capabilities for cellulose [41,42], while the *T. longibrachiatum* strain A25011 was demonstrated to have specific advantages in chitosan decomposition. In peanut seedling trials, *T. longibrachiatum* A25011 showed growth-promoting effects [43]. New research showed that the cellulase of *Trichoderma* not only directly inhibited the growth of pathogens, but also prevented and controlled *Fusarium* stem rot by activating plant host defense mechanisms [44,45].

HPLC-MS/MS analysis identified a complete hormone system in *T. longibrachiatum* A25011, including higher concentrations of GA3, CZR, and IAA. The concentration of GA3 in A25011 can promote seed germination within the physiological activity range, stimulate cell division and elongation, significantly enhance plant height, and promote cell elongation and flowering [46,47,48]. Both CZR and TZR are core bioactive cytokinins (CKs), and TZR exhibits higher biological activity, as proven by Stirk [49]. Zeatin riboside functions primarily in maintaining leaf chlorophyll and protein synthesis, maintaining chlorophyll cycle and photosynthetic complexes, counteracting ABA-induced senescence, and participating in stress responses and signal transduction [50,51]. IAA can stimulate cell elongation and organ differentiation, contrasting with its senescence-accelerating effects at higher concentrations [52]. ABA, present at low levels (0.06 mg/L), enhances stress resistance without triggering the germination inhibition or organ abscission observed at elevated concentrations [53,54]. KT was detected at minimal levels, suggesting only synergistic roles in this strain. Much research has also indicated that *Trichoderma* has a relatively complete hormone system, which has a positive impact on the germination of plant seeds, the growth of stems and leaves, and, ultimately, flowering and fruit ripening [55,56]. The concentrations of these hormones in *T. longibrachiatum* A25011 fall within the physiological range for growth promotion, synergistically enhancing plant growth (mainly root weight and plant height) and explaining the observed 28-day growth enhancement phenomenon in peanut (Figure 5). In future work, we will evaluate the effects of *T. longibrachiatum* A25011 on seed germination, plant growth, and biocontrol efficacy against pathogens across different crops.

Furthermore, the inhibitory effect of *T. longibrachiatum* A25011 on fumonisins has positive implications for food safety. A strong positive correlation (r = 0.817) between mycotoxin amount and *F. proliferatum* DNA biomass was proven in our previous study [57]. Therefore, the fumonisin concentration in rice samples corresponds to the *F. proliferatum* DNA biomass. Previous research shows that co-cultivation of *Trichoderma* with *Fusarium* significantly reduced the pathogen’s ability to produce FB1 in colonization substrates, a finding consistent with our research [58]. This discovery transcends traditional biocontrol outcomes and provides a novel perspective on addressing widespread crop fungal contamination issues through mycotoxin mitigation.

However, because the growth environment of *Trichoderma* can vary considerably, the extracellular enzyme and hormone profile of *T. longibrachiatum* A25011 is likely to fluctuate, highlighting the need for further experiments under diverse culture conditions. Additionally, the inhibitory mechanism of *T. longibrachiatum* A25011 on fumonisin accumulation requires an in-depth investigation to clarify whether the observed effects result from the suppression of mycotoxin biosynthesis or from the degradation of fumonisins already produced. Our understanding of crop–microbe interactions relies on experiments conducted in simplified laboratory settings, which often fail to translate directly into the complex and dynamic field environment. Consequently, when designing microbial biocontrol agents, we must account for interactions and persistence within existing soil microbiomes, the trade-offs between growth promotion function, community stability under host plant and environmental regulation, and meticulous investigation of biocontrol agent delivery methods and carrier systems [59].

Future research should prioritize field trials under varying soil pH values, temperatures, and humidity conditions to assess the robustness of *T. longibrachiatum* A25011 as a biological control agent, especially in crops susceptible to *Fusarium* infections. The comprehensive study of *T. longibrachiatum* A25011 and its interactions with indigenous soil microbiota and crop plants requires integrating multi-omics data, including genomics, transcriptomics, and metabolomics. Meanwhile, cross-validation using transcriptomics and metabolomics can be employed to verify whether *T. longibrachiatum* A25011 has the ability to degrade fumonisins and further investigate the active substances responsible for its efficient fumonisin degradation. Additionally, exploring the application of *T. longibrachiatum* A25011 in combination with other microorganisms as biocontrol agents can leverage complementary antimicrobial spectra and also enhance the biological effects to guarantee food security [60].

In conclusion, *T. longibrachiatum* A25011 efficiently inhibited *Fusarium* growth and fumonisins accumulation, while also demonstrating the capacity to enhance crop protection, promote plant growth, and improve food safety. Its commercial development holds significant potential for advancing sustainable agriculture.

## Figures and Tables

**Table 1:** Mass spectrometric parameters and calibration data for the quantification of target hormones.

Hormones	Parent Ion m/z	Daughter Ion m/z	Linear Range	R^2^
Indole-3-acetic Acid (IAA)	176.07	76.92/88.92	0.1–200 ng/mL	0.9998
Kinetin (KT)	215.93	80.97/147.83	0.1–200 ng/mL	0.9985
cis-Zeatin Riboside (CZR)	352.16	136.00/185.00	0.1–200 ng/mL	0.9926
Gibberellin A3 (GA3)	345.13	143.08/221.08	0.1–200 ng/mL	0.9991
Abscisic Acid (ABA)	263.13	153.00/203.00	0.1–200 ng/mL	0.9986
trans-Zeatin Riboside (TZR)	352.16	202.00/220.00	0.1–200 ng/mL	0.9941

**Table 2:** Concentration of various hormones produced by *T. longibrachiatum* strain A25011 after 7 days of culture on PDA plates.

Hormones	GA3	CZR	IAA	ABA	KT	TZR
content (mg/L) *	2.44 ± 0.04	0.68 ± 0.01	0.35 ± 0.02	0.06 ± 0.01	0.006 ± 0.001	0.004 ± 0.001

* Means and standard deviations (S.D.s) are given in the table (n = 12).

**Table 3:** Enzyme activity of chitinase and moderate cellulase produced by *T. longibrachiatum* strain A25011 after 7 days of culture on PDA plates.

Enzymes	Chitinase	Cellulase
Enzyme activity (units/mg protein) *	583.21 ± 4.00	43.92 ± 1.18

* Means and standard deviations (S.D.s) are shown in the table (n = 24).

## Data Availability

The corresponding author can provide the data backing up these conclusions upon reasonable request.

## Abbreviations

The following abbreviations are used in this manuscript:

BLAST	Basic local alignment search tool
HPLC-MS/MS	High-performance liquid chromatography with tandem mass spectrometry
GA3	Gibberellin A3
CZR	cis-Zeatin riboside
TZR	trans-Zeatin riboside
IAA	Indolacetic acid
DON	Deoxynivalenol
ZEN	Zearalenone
ELEM	Equine leukoencephalomalacia
BCAs	Biological control agents
PDA	Potato dextrose agar
DNA	Deoxyribonucleic acid
CTAB	Cetyl trimethyl ammonium bromide
ITS	Internal transcribed spacer
TEF	Translation elongation factor
PCR	Polymerase chain reaction
